# Enhancing fetal outcomes in GCK-MODY pregnancies: a precision medicine approach via non-invasive prenatal *GCK* mutation detection

**DOI:** 10.3389/fmed.2024.1347290

**Published:** 2024-04-30

**Authors:** Valérie M. Schwitzgebel, Jean-Louis Blouin, Barbara Dehos, Bettina Köhler-Ballan, Jardena J. Puder, Claudine Rieubland, Maria Triantafyllidou, Anne Zanchi, Marc Abramowicz, Thierry Nouspikel

**Affiliations:** ^1^Pediatric Endocrine and Diabetes Unit, Department of Pediatrics, Gynecology and Obstetrics, Geneva University Hospitals, Geneva, Switzerland; ^2^Diabetes Center of the Faculty of Medicine, University of Geneva, Geneva, Switzerland; ^3^Genetic Medicine, Diagnostic Department, Geneva University Hospitals, Geneva, Switzerland; ^4^Department of Genetic Medicine and Development, Faculty of Medicine, University of Geneva, Geneva, Switzerland; ^5^Division of Endocrinology and Diabetes, Spital Grabs, Grabs, Switzerland; ^6^Department of Infectious Disease, Geneva University Hospitals, Geneva, Switzerland; ^7^Department Women-Mother-Child, Lausanne University Hospital and University of Lausanne, Lausanne, Switzerland; ^8^Department of Medical Genetics, Central Institute of the Hospitals, Hospital of the Valais, Valais, Switzerland; ^9^Division of Endocrinology, Diabetes and Clinical Nutrition, Luzerner Kantonsspital, Lucerne, Switzerland; ^10^Department of Medicine, Service of Endocrinology, Diabetes and Metabolism, Lausanne University Hospital, Lausanne, Switzerland

**Keywords:** Non-invasive prenatal diagnosis, monogenic diabetes, GCK-MODY, cell-free circulating DNA, fetal DNA, glucokinase, *in utero* therapy, personalized medicine

## Abstract

**Background:**

Mutations in the *GCK* gene cause Maturity Onset Diabetes of the Young (GCK-MODY) by impairing glucose-sensing in pancreatic beta cells. During pregnancy, managing this type of diabetes varies based on fetal genotype. Fetuses carrying a *GCK* mutation can derive benefit from moderate maternal hyperglycemia, stimulating insulin secretion in fetal islets, whereas this may cause macrosomia in wild-type fetuses. Modulating maternal glycemia can thus be viewed as a form of personalized prenatal therapy, highly beneficial but not justifying the risk of invasive testing. We therefore developed a monogenic non-invasive prenatal diagnostic (NIPD-M) test to reliably detect the transmission of a known maternal *GCK* mutation to the fetus.

**Methods:**

A small amount of fetal circulating cell-free DNA is present in maternal plasma but cannot be distinguished from maternal cell-free DNA. Determining transmission of a maternal mutation to the fetus thus implies sequencing adjacent polymorphisms to determine the balance of maternal haplotypes, the transmitted haplotype being over-represented in maternal plasma.

**Results:**

Here we present a series of such tests in which fetal genotype was successfully determined and show that it can be used to guide therapeutic decisions during pregnancy and improve the outcome for the offspring. We discuss several potential hurdles inherent to the technique, and strategies to overcome these.

**Conclusion:**

Our NIPD-M test allows reliable determination of the presence of a maternal *GCK* mutation in the fetus, thereby allowing personalized *in utero* therapy by modulating maternal glycemia, without incurring the risk of miscarriage inherent to invasive testing.

## Introduction

The *GCK* gene encodes glucokinase, a key enzyme for glucose sensing by pancreatic beta cells. Patients with a heterozygous mutation in *GCK* present with Maturity Onset Diabetes of the Young (GCK-MODY) since their beta cells, underestimating glycemia, fail to release adequate amounts of insulin (1). In pregnancies where the mother carries a *GCK* mutation, it is important to rapidly determine whether the fetus inherited the maternal mutation since treatment recommendations differ from other types of gestational diabetes (1, 2). If the fetus carries the mutation, moderate maternal hyperglycemia is beneficial, as it promotes fetal insulin secretion in the fetus and prevents intra-uterine growth retardation (2, 3). For a fetus bearing the wild-type allele, however, maternal hyperglycemia should be avoided since it may cause macrosomia (4).

Modulating maternal glycemia thus constitutes a form of natural *in utero* therapy, which is highly beneficial for the fetus, yet does not justify the risk of invasive prenatal testing to determine fetal genotype. Our laboratory recently developed a non-invasive prenatal diagnostic test for monogenic diseases (NIPD-M), which relies on the presence of circulating cell-free DNA (ccfDNA) in maternal plasma (5). Since cell-free fetal DNA (cffDNA) only represents a small fraction of total ccfDNA and cannot be distinguished from maternal ccfDNA, determining whether the fetus inherited the maternal mutation is particularly challenging. The allelic balance at the mutation site is expected to be 50:50 when a heterozygous mother carries a heterozygous fetus, and to deviate from this ratio in case the fetus is homozygous for the wild-type allele. A test relying on this principle, Relative Mutation Dosage (RMD), has been described (6, 7) but is rarely used in the clinics since the extremely low amounts of cffDNA in maternal plasma make it difficult to gather enough molecular counts from a single genomic position to reach statistical significance. An elegant solution, Relative Haplotype DOsage (RHDO), includes multiple heterozygous single nucleotide polymorphisms (SNPs) adjacent to the mutation in the calculation of a haplotype balance (9). The maternal haplotype inherited by the fetus is expected to be overrepresented in total ccfDNA and, provided the phase of each SNP in relation to the mutation has been determined in advance, one can ascertain whether the fetus inherited the high-risk (mutated) or the low-risk (wild-type) haplotype (cf. Supplementary Figures 1, 2).

The applicability of this method to GCK-MODY has been demonstrated by us (5) and others (7, 8) in proof-of-concept studies. In the present study, we showcase a series of NIPD-M analyses conducted on pregnant women who are heterozygous carriers of a *GCK* mutation. We describe the challenges encountered and the solutions implemented, that enabled diagnostic results for the participating couples.

## Results

Our NIPD-M series includes 10 samples from 7 pregnant women known to carry *GCK* mutations whereas their respective partners did not (one mother had 3 pregnancies and donated two samples from the first). Table 1 summarizes our results, which revealed that five fetuses carried the maternal mutation, and four did not. For all prospective cases, we were able to deliver test results within 4–11 days (9 days on average).

**TABLE 1:** Test results and clinical data.

Sample	Origins	*GCK* mutation	Weeks	ccfDNA (ng)	FF	SNPs	DNA molecules	Genotype	Likelihood	Treatment	Target glycemia	Monitoring	Term (weeks)	Weight (g) centile	Gender	Remarks
Family 1, child 1, sample 1	Caucasian	c.736G>A, p.Gly246Arg	12	7.2	8.9%	17	3,733	Carrier	1.72E+09	None	Fasting 5.8–7.1 mmol/L 1 h post-prandial <8.5 mmol/L	HbA1C 5.6–6.3%	38 6/7	3,240 (P50)	Female	Retrospective. Two samples received at different times.
Family 1, child 1, sample 2			30	15	19.4%	17	2,209	Carrier	9.96E+22							
Family 1, child 2			22 1/7	15.3	15.9%	17	4,002	Carrier	1.41E+18	None			37 6/7	3,045 (P50)	Female	Retrospective. Second pregnancy of same patient.
Family 1, child 3			18 3/7	19.6	18.2%	17	3,663	Carrier	7.76E+21	None	Fasting 5.8–7.1 mmol/L 1 h post-prandial <8.5 mmol/L	HbA1C 5.6–6.3%	38 4/7	3,053 (P75–P90)	Male	Third pregnancy of same patient. APGAR 9/10/10.
Family 2	Italy	c.834C>A, p.Asp278Glu	28	10.1	15.5%	5	407	Carrier	2243	Insulin analogs at mealtimes			37 3/7	2,620 (P10–P25)	Female	Retrospective. Delivered by C-section. 45 cm, APGAR 9/9/10. RDS after birth.
Family 3, sample 1	Spain	c.227C>T, p.Ser76Phe	14	9	–	22	–	Test failed	–	Insulin analogs at mealtimes	Fasting ≤5.3 mmol/L 2 h post-prandial <7.0 mmol/L	HbA1C 5.5–5.8%	39	2,925 (P10–P25)	Female	48 cm, APGAR 10/10/10. No hypoglycemia at birth.
Family 3, sample 2			21	14.4	11.4%	22	1,925	Wild-type	6.05E+04							
Family 4	Switzerland	c.991G>T, p.Glu331	28	15	17.8%	15	1,145	Wild-type	1.30E+09	Insulin at bedtime and mealtimes from 29 weeks	Fasting ≤5.3 mmol/L 1 h post-prandial ≤8.0 mmol/L	Always in range	38 5/7	2,670 (P10)	Female	47 cm, APGAR 9/10/10.
Family 5	Switzerland	c.608T>C, p.Val203Ala	14 4/7	50	10.7%	11	1,155	Wild-type	1.56E+06	Insulin at mealtimes from 18 weeks	Fasting ≤5.3 mmol/L 1 h post-prandial ≤8.0 mmol/L	70–80% in range (3.9–8.0 mmol/L)	40	3,330 (P25–P50)	Female	49 cm, APGAR 10/10/10. Mother may also have GDM
Family 6	Caucasian	c.106C>T, p.Arg36Trp	15	40	11.1%	9	2,949	Carrier*	4.61E−09	None	Fasting ≤5.3 mmol/L 1 h post-prandial ≤8.0 mmol/L	Morning glycemia 5–6 mmol/L Post-prandial <8 mmol/L	38 2/7	3,770 (P75–P90)	Male	51 cm, APGAR 10/10/10.
Family 7	Switzerland	c.608T>C, p.Val203Ala	16	50	16.0%	15	7,363	Wild-type	1.35E+26	None so far	–	–	–	–	–	Ongoing pregnancy.

Note added after acceptance of the article and before publication: We recently obtained evidence that family 6 had a vanishing twin pregnancy and that the surviving twin did not carry the mutation.

Three of these tests were performed as part of our method validation procedure and these results were not used for clinical decisions. Nevertheless, fetal genotype was predicted from maternal plasma, and the children were genotyped after birth, retrospectively confirming that these predictions were accurate.

For fetuses shown early in pregnancy to carry the maternal mutation, our strategy was to allow moderate maternal hyperglycemia. Fetal growth was normal, and these children were born at term, with normal weight and size and normal post-natal glycemia.

For fetuses that did not inherit the maternal mutation, we aimed at maintaining maternal glycemia within the recommended limits for gestational diabetes: fasting blood sugar ≤5.3 mmol/L, 1 h postprandial ≤8.0 mmol/L, or 1–2 h postprandial ≤7.0 mmol/L. Details of the treatment can be found in Table 1 and Supplementary material. Fetal growth was normal and three of the children were born at term with no indication of macrosomia. One pregnancy is still ongoing at the time of writing.

**FIGURE 1:**
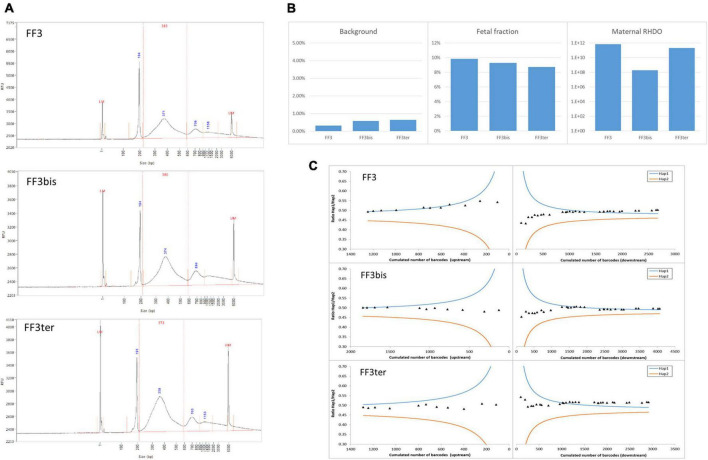
Repeatability and reproducibility. The same ccfDNA sample was tested three times, twice in parallel (FF3 and FF3bis) and once on a different day (FF3ter). **(A)** DNA size profiles for the three libraries. The larger peaks (>600 bp) are migration artifacts due to secondary structures and do not impact sequencing. **(B)** Sequencing background, estimation of FF, and likelihood ratio for the RHDO analysis. **(C)** Crossing-over exclusion graphs. The mutation is in the center and SNPs (diamonds) are plotted in order of appearance on either side. *X*-axis: cumulated molecular counts for this SNP and all the previous SNPs. *Y*-axis: allelic ratio for the cumulated molecules. The curves represent the 1,200:1 diagnostic threshold for haplotype 1 (blue) and 2 (orange).

To investigate the repeatability and reproducibility of our NIPD-M test, we analyzed the same sample three times, once in duplicate, once on a different day. Figure 1 shows that the results, including various quality control metrics, were highly similar in all three cases.

**FIGURE 2:**
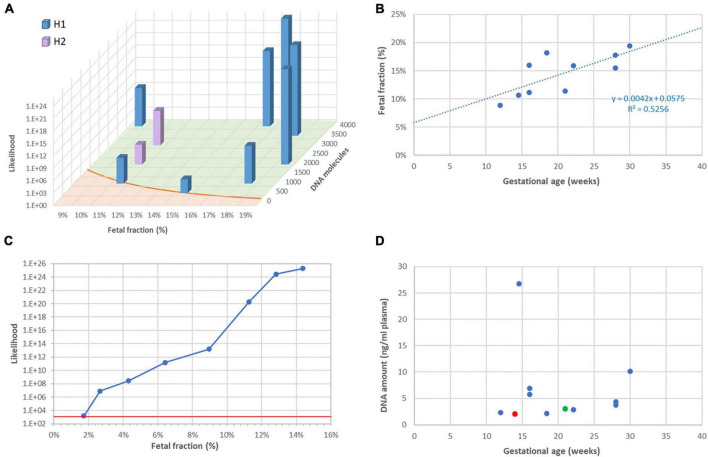
Fetal fraction and molecular counts. **(A)**: Dependency of result likelihood on FF and molecular counts. The red line represents the theoretical diagnostic threshold 1,200:1. **(B)** Correlation between FF and gestational age. **(C)** Artificial reduction of FF by mixing sequencing data from the plasma with data from the mother. The red line represents the diagnostic threshold 1,200:1. **(D)** Lack of correlation between ccfDNA amount in plasma and gestational age. The red symbol represents one case for which library construction failed, which required resampling (green symbol).

It is well established that the reliability of RHDO analyses, specifically the likelihood ratio between the most likely and the less likely haplotype, largely depends on two factors: the fetal faction (FF) and the number of DNA molecules tallied. The latter in turn depends on the efficiency of the library system, the initial amount of ccfDNA in the reaction, and most importantly the number of informative SNPs for a given couple. This is indeed what we observed in this series, and likelihood ratios were in accordance with the theoretical model (Figure 2A).

As described by others (10), we observed that FF tends to increase during pregnancy (Figure 2B), implying that a later test has greater chances of success. To further investigate the dependency of the likelihood ratio toward FF, we artificially reduced FF in a sample in which it originally was 14.4%, by mixing sequencing data from plasma with sequencing data from maternal leukocytes. Figure 2C shows that the significance threshold of 1,200:1 can still be reached with FF as low as 2%.

Contrarily to FF, the concentration of ccfDNA in plasma was not correlated with gestational age (Figure 2D). This is not surprising, as it is known to be influenced by multiple individual factors, such as body mass index (11), physical exercise (12), and even psycho-social stress (13). In our series, ccfDNA yield varied between 2.0 and 26.8 ng/ml plasma. On one occasion (family 3), this resulted in a total amount of only 9 ng DNA and library building was unsuccessful. We repeated sampling a few weeks later, obtained a higher amount of DNA and were able to return a diagnostic result. After this incident, we decided to routinely ask for 20 ml blood instead of 10 ml, to reduce the risk of test failure due to insufficient DNA.

This strategy is however limited by the amount of blood that can reasonably be requested for a routine test. Thus, in practice, the number of DNA fragments available for analysis mostly depends on the number of informative SNPs. RHDO analysis is based on “type-4” SNPs (Supplementary Figures 1, 2), for which the mother is heterozygous, so haplotypes can be distinguished, and the father is homozygous, so the paternal contribution to fetal genotype is known (9). It is, however, impossible to predict how many such SNPs will be available for a given couple.

**FIGURE 3:**
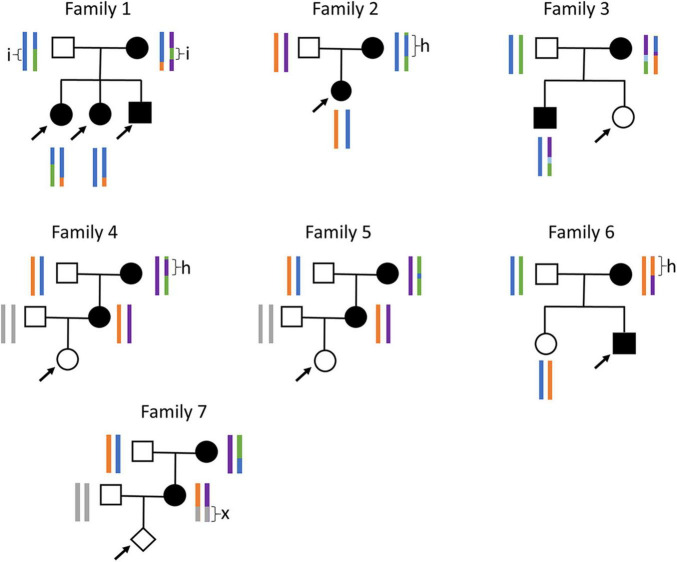
Families and haplotypes. Family trees and haplotypes (color bars) for the seven families tested. h, stretches of homozygosity; i, stretch of biallelic identity-by-state; x, stretch that could not be phased since both grandparents are heterozygous. Arrows: fetuses tested by NIPD-M. Only haplotypes available prior to plasma testing are shown. For families 4, 5, and 7, with no previous child available, maternal haplotypes were reconstructed using the grandparents and paternal haplotypes were not determined.

There seem to be several common *GCK* haplotypes in the European population, as we occasionally observed large stretches of homozygosity in the *GCK* region (Figure 3). When this happens in the mother, it drastically reduces the number of type-4 SNPs available for analysis. We encountered this problem with family 2, in which the mother carries a 365 kb stretch of homozygosity around the *GCK* gene, resulting in only five informative SNPs in the entire region. Our initial analysis with 259 DNA molecules failed to reach significance, with a likelihood ratio of 605:1, thus inferior to the recommended threshold of 1,200:1 (9). Since we had a small amount of ccfDNA left, we repeated the test, which yielded the same result with a likelihood of 4:1 for 148 molecules. However, pooling molecular counts from both experiments allowed reaching the diagnostic threshold, with a likelihood of 2,243:1.

Another risk when frequent haplotypes exist in a population is that the parents may share stretches of identity-by-state. When this happens for both haplotypes, such a stretch is entirely devoid of type-4 SNPs. We encountered this situation for family 1, which carries a 200 kb stretch of biallelic identity-by-state in the *GCK* locus. Fortunately, there were enough type-4 SNPs outside this stretch to allow diagnosis. When this is not the case, we have shown that it is possible to leverage type-5 SNPs, for which both parents are heterozygous, to directly determine fetal genotype (Supplementary Figure 2). We initially developed this approach, Relative Genotype DOsage (RGDO), for highly consanguineous couples, who often carry large stretches of biallelic identity-by-descent (14), but it can also be used in non-consanguineous couples to overcome stretches of biallelic identity-by-state, and we have successfully applied it to family 1 to reinforce the conclusions obtained with RHDO (5).

The use of heterozygous SNPs, whether type-4 or type-5, implies a requirement to phase them into haplotypes, i.e., to determine for each SNP which allele belongs to the mutated and which to the wild-type haplotype. This is usually achieved by genotyping a prior child of the consulting couple, but may not always be possible, for instance when it is the first pregnancy of a couple. We encountered such a situation with families 4, 5, and 7, but in all cases we were able to phase most of the SNPs by genotyping the maternal grandparents (Supplementary Figure 3). However, statistically, around 25% of the SNPs are expected to be heterozygous in both grandparents, making them impossible to phase in the mother. Indeed, we were only able to phase 35 SNPs out of 53 (66%) for family 4, 30 SNPs out of 51 (59%) for family 5, and 15 out of 45 (33%) for family 7, which nevertheless proved sufficient to obtain diagnostic results for all 3 families. When this is not the case, or when the grandparents (or other first-degree relatives) are not available, several phasing strategies can be envisioned to reconstitute haplotypes: long-read sequencing (14), targeted locus amplification phasing (15), or barcoded linked-read sequencing (16).

A potential problem with all techniques relying on haplotype determination is the possibility of a meiotic crossing-over within the region examined. Such an event is revealed by the detection of haplotype 1 on one side of the mutation and haplotype 2 on the other, but uncertainty on the exact location of the crossing-over will likely make it impossible to determine which haplotype is present at the mutation site in the fetus. To exclude crossing-overs, results are plotted on a bidirectional graph, starting from the mutation, and cumulating molecular counts for all SNPs successively encountered in either direction (9) (Supplementary Figure 4). This implies that the likelihood threshold must be reached on each side of the mutation, which may not always be possible, depending on the repartition of informative SNPs in the analyzed region.

We encountered such a difficulty in families 2, 4, 5, and 6, in which the maternal haplotype transmitted to the fetus was unequivocally determined on one side of the mutation, whereas the likelihood threshold was not reached on the other. To resolve these situations, we first calculated the probability of a crossing-over occurring between the mutation and the first diagnostic SNP on the informative side, then combined it with the likelihood for the opposite haplotype on the other side (see Supplementary Figure 5 for details). Using this method, we obtained haplotype likelihood ratios of 911:1 for family 2, 23,391:1 for family 4, 29,929:1 for family 5, and 32,216:1 for family 6. With the usual cutoff of 1,200:1, this strategy thus resulted in a non-informative result for family 2 (retrospective test), and in diagnostic results for the other 3 families.

## Discussion

Here we show that our NIPD-M test applied to *GCK* mutations proficiently discerns the fetal genotype as early as 12 weeks of amenorrhea. Such timely determination facilitates a personalized therapeutic strategy based on fetal genotype. In instances where the fetus has inherited the maternal mutation, a moderate elevation in maternal glucose proves beneficial by augmenting fetal insulin secretion and glucose utilization. Within our study cohort, there were no instances of intra-uterine growth retardation or suboptimal birth weight. Conversely, for fetuses inheriting the wild-type allele, it is imperative to maintain normoglycemia due to the potential risk of macrosomia associated with hyperglycemia. Again, there were no indications of macrosomia in our cohort, and all children had normal birth weight.

We also describe several hurdles that may be encountered with this technique and suggest strategies to overcome them. The typically low abundance of ccfDNA in plasma, which results in low molecular counts, can be remediated by drawing more blood (we suggest 20 ml) or by repeating the test at a later time and pooling data. Similarly, an excessively low FF may require later resampling, as FF is known to increase during pregnancy. Yet fetal genotype information should be available as early as possible so fetal wellbeing can be optimized early on. Based on the results presented here and our experience with other genes, we recommend performing the initial test around 12–14 weeks of amenorrhea.

The fact that RHDO is entirely based on SNP genotyping has the advantage that it can be used to interrogate mutations that are not detectable by sequencing, for instance large deletions or chromosomal rearrangements disrupting the gene of interest. The drawback of this reliance on SNPs is that the required number of informative SNPs may not always be reached for a given couple. In particular, the existence of common haplotypes in the general population may result in large stretches of homozygosity in the mother, or in biallelic regions of identity between the parents.

We have shown that the latter situation can be solved using RGDO, a variant of RHDO which we originally designed for consanguineous couples (14). Long stretches of homozygosity are more difficult to deal with, as diagnosis can only be achieved with heterozygous SNPs located outside this stretch. A possible strategy would thus be to design a SNP panel encompassing a larger genomic region, thereby minimizing the impact of a stretch of homozygosity. One should keep in mind, though, that a larger panel implies a greater risk of crossing-over. In our experience with *GCK* and other genes, we found that panels spanning 1 Mb are generally a good compromise.

Ruling out the presence of a crossing-over is essential for these analyses but requires not only that there are sufficient informative SNPs, but also that these SNPs are distributed on both sides of the mutation. When this is not the case, it may happen that the diagnostic threshold is not reached on one side of the mutation. We propose a strategy to resolve such situations by including the probability of recombination in likelihood calculations. Using this approach, we were able to resolve three out of four such cases.

The remaining case, family 2, was investigated retrospectively during method validation and there was therefore no requirement to provide a diagnostic result. Had this been the case, we would have pondered whether a likelihood of 911 against 1 was sufficient for therapeutic decision. This is unlikely with severe genetic conditions when pregnancy termination is considered. In the case of GCK-MODY, however, the goal is to optimize fetal wellbeing and the endocrinologist in charge of the couple concluded that a probability of 911 against 1 for the fetus to carry the mutation would have been sufficient to orient therapy. Furthermore, it is always possible to monitor pregnancy for early signs of macrosomia (1).

Finally, a limitation of RHDO is that it requires a prior conceptus of the couple to properly phase parental haplotypes. When such a sample is not available, we show that one can successfully resort to genotyping the grandparents or a first-degree relative of the person carrying the mutation. This, however, implies a drop in efficiency since it generally will not be possible to phase all informative SNPs. There are several technical alternatives to ensure complete phasing and we have shown that long-read sequencing is an efficient option, although it nearly doubles the cost of the test (5).

While this manuscript was in preparation, Hughes et al. (17) reported on a series of 38 cases investigated by RMD, with digital PCR. The RMD approach is highly challenging as it tests a single genomic position, and this probably explains the relatively high rates of inconclusive tests (5/43) and incorrect diagnosis (1/38) reported by the authors. However, errors have less drastic consequences in the context of GCK-MODY, and the authors convincingly demonstrate that a PPV of 96% is far better that what can be achieved with the traditional method of detecting macrosomia by echography (measurement of fetal abdominal circumference).

Hughes et al. (17) also studied the feasibility of implementing a NIPD-M approach for *GCK* in the context of the British National Health Service and identified two potential obstacles: the cost of the test and a relatively long turnaround time. Both likely derive from the fact that RMD is a mutation-specific test, which implies designing and manufacturing a new test for potentially every new patient. In particular, the authors report a turnaround time of 2 weeks for a mutation test already established in their laboratory, versus 7 weeks for a novel mutation. By contrast, RHDO is gene-specific and relies on common SNPs, so the same reagents can be used for all patients. As a result, our average turnaround times is only 9 days and, while Hughes et al. (17) quote a price of 2,000 GBP for their test, we bill ours almost 3 times less, 920 CHF, which is acceptable for the Swiss system of mandatory subscription to a private health insurance.

In conclusion, this small series demonstrates that NIPD-M applied to the GCK locus is a robust and reliable test that allows, with no risk of iatrogenic miscarriage, the proper installment of a personalized prenatal therapy, by modulating maternal glycemia to optimize fetal wellbeing.

## Materials and methods

Samples were processed as previously described (5). Briefly, maternal venous blood was collected into Streck BCT tubes (Streck) between 12 and 30 weeks amenorrhea. Plasma was prepared by centrifugation for 10 min at 1,600 × *g*, 4°C, collected and centrifuged 10 min at 16,000 × *g*, 4°C. Circulating cell-free DNA was extracted from plasma with the QIAamp MinElute ccfDNA kit (Qiagen) according to the manufacturer’s instructions.

QIASeq targeted libraries were built according to the manufacturer’s protocol, using 40 ng genomic DNA or 7.2–50 ng ccfDNA, then sequenced as 2 × 75 nucleotides with a NextSeq500 sequencer (Illumina).

Qiagen smCounter2 pipeline was used to align and filter reads, deduplicate barcodes and build consensus reads using Fgbio subroutines.^1^ Allele counts were extracted with bam-readcount.^2^ RHDO was performed in Excel, as described by Lo et al. (9). Type-4 SNPs (mother heterozygous and father homozygous) were separated into alpha (father homozygous for maternal haplotype 1) and beta (father homozygous for maternal haplotype 2). Alpha and beta SNPs located upstream and downstream of the mutation were plotted separately, to exclude crossing-overs within the region of interest. A global likelihood ratio was then calculated for all alpha SNPs or for all beta SNPs.

See Supplementary material for additional method details.

## Data availability statement

The datasets presented in this study can be found in online repositories. The names of the repository/repositories and accession number(s) can be found below: https://www.ncbi.nlm.nih.gov/bioproject/PRJNA1048410.

## Ethics statement

Written informed consent was obtained from the individual(s) for the publication of any potentially identifiable images or data included in this article.

## Author contributions

VS: Resources, Writing – review & editing. J-LB: Funding acquisition, Investigation, Supervision, Writing – review & editing. BD: Resources, Writing – review & editing. BK-B: Resources, Writing – review & editing. JP: Resources, Writing – review & editing. CR: Resources, Writing – review & editing. MT: Resources, Writing – review & editing. AZ: Resources, Writing – review & editing. MA: Funding acquisition, Supervision, Writing – review & editing. TN: Conceptualization, Investigation, Methodology, Supervision, Writing – original draft, Writing – review & editing.

## Footnotes

1
https://github.com/fulcrumgenomics/fgbio

2
https://github.com/genome/bam-readcount

## Supplementary material

Supplementary Table 1 List of SNPs in the GCK panel.

Supplementary Data Sheet 1 Detailed clinical history, detailed methods, legends to supplementary figures.

Supplementary Figure 1 Type of SNPs and their usage.

Supplementary Figure 2 Principles of RHDO and RGDO.

Supplementary Figure 3 Analytical workflow.

Supplementary Figure 4 Crossing-over detection.

Supplementary Figure 5 Calculations when crossing-over is not excluded.
