# Supplementary material for: Enhancing fetal outcomes in GCK-MODY pregnancies: a precision medicine approach via non-invasive prenatal GCK mutation detection
Source: Front Med (Lausanne). 2024 Apr 30;11:1347290. doi: 10.3389/fmed.2024.1347290 (PMC11091329; doi:10.3389/fmed.2024.1347290)
Supplement: Supplementary Data Sheet 1 — Detailed clinical history, detailed methods, legends to supplementary figures. [file Data_Sheet_1.docx]

**Contents**

1. Detailed clinical history……………………………………………………………………………………………………………. 1
2. Detailed methods……………………………………………………………………………………………………………………. 4
3. Figure legend………………………………………………………………………………………………………………………….. 6

**1. DETAILED CLINICAL HISTORY**

Family 1. Fetus 1, carrier

This pregnancy was part of our validation cohort, thus information on fetal genotype was not used for clinical decisions. The mother did not receive insulin treatment but during the entire pregnancy her fasting glycemia remained in the range 5.8-7.1 mmol/l, and inferior to 8.5 mmol/l at 1h post-prandial. HBA1c values ranged between 5.6% and 6.3%. The pregnancy was also carefully monitored for signs of macrosomia.

A baby girl was born at 38 6/7 weeks with a birth weight of 3.24 kg (p25-50). DNA was extracted from cord blood to serve as surrogate “previous child” for retrospective determination of parental haplotypes, as well as to subsequently validate NIPD-M results.

Family 1. Fetus 2, carrier

This second pregnancy from the same couple was also part of the validation cohort and was handled as above.

A baby girl was born at 37 6/7 weeks with a birth weight of 3.045 kg (P50). DNA was extracted from cord blood to validate NIPD-M results.

Family 1. Fetus 3, carrier

This was the third pregnancy from the above couple. At this time, our method was validated, and the test was performed in a diagnostic context. As the test revealed that the fetus carried the maternal mutation, the mother did not receive insulin treatment but was monitored throughout her pregnancy. Her fasting glycemia values remained in the range 5.8-7.1 mmol/l, and inferior to 8.5 mmol/l, 1h post-prandial, HBA1c was 5.6-6.3%. She gained 7-8 kg during pregnancy, without any complication.

A baby boy was born at 38 4/7 weeks, with a birth weight of 3.53 kg (p75-90), APGAR scores were 9/10/10. Postnatal confirmation of the familial *GCK* mutation is in the planning.

Family 2. Carrier fetus

This pregnancy was part of our test validation cohort, thus information on fetal genotype was not used for clinical decisions. The mother was treated with insulin analogs at mealtimes: 5-16 U Humalog as needed at the end of pregnancy. The pregnancy was carefully monitored for signs of IUGR.

A baby girl was delivered by C-section at 37 3/7 weeks with normal birth weight (2.62 kg, P10-P25) and size (45 cm). APGAR scores were 9/9/10, arterial pH 7.25, venous pH 7.39. The newborn subsequently presented with RDS and had to be briefly hospitalized in the neonatology unit. She was later genotyped for confirmation of the prenatal results.

Family 3. Wild-type fetus

The mother was a known carrier of the *GCK:*c.227C>T, p.Ser76Phe mutation, which was diagnosed in the research laboratory where she worked. For the present pregnancy, the mother was treated with insulin analogs Lantus and Novorapid, administered prior to meals. Targeted fasting glycemia was <5.3 mmol/L, 2h postprandial glycemia <7 mmol/L. Glycemia was monitored with a Freestyle Libre 2 sensor and well controlled during pregnancy, with HbA1C measured at 5.5% to 5.8%.

A female child was born at 39 weeks, with normal birth weight (2.925 kg, P10-P25) and size (48 cm). APGAR scores were 10/10/10. No hypoglycemia at birth.

Family 4. Wild-type fetus

The patient was a 31-year-old woman, with a pathological OGTT in the 18th week of gestation. The test was run due to positive family history (the patient's mother had NIDDM, diagnosed a long time ago, always rather slim, and very well controlled under Metformin monotherapy). The patient herself was not overweight but was referred to us with a HbA1c of 6.6% in the 18th week of gestation. Due to the highly suspected MODY-GCK we decided, together with the patient and her gynecologist, to follow a watchful waiting strategy, with regular capillary blood sugar measurements and monthly fetal ultrasounds. After confirmation of the diagnosis, we continued with the same regimen. Capillary measurements never exceeded 8 mmol/l fasting or 11 mmol/ l pp and fetal growth remained below 50th percentile. The normal fetal NIPD-M result came about simultaneously with a growth spurt at above 70th percentile where we began with an intensive insulin therapy, at bedtime and prandially for the rest of the pregnancy. As of this point, glycemic targets were the same as for all gestational diabetes patients (fasting ≤ 5.3 mmol/l and 1h pp ≤ 8.0 mmol/l). Our patient was very compliant and always on target.

She gave birth vaginally to a girl, at 38 weeks and 5 days. Birth weight was 2670g (P10), height 47cm, head circumference 33cm, APGAR score 9 / 10 / 10. There was no other significant event during pregnancy or after the birth of the child.

Family 5. Wild-type fetus

Targeted glycemia followed the recommended values for GDM (fasting blood sugar ≤ 5.3mmol/l, 1h postprandial ≤ 8.0 mmol/l, 2h postprandial ≤ 7.0 mmol/l). The patient was monitored with capillary measurements, which surprisingly showed not only the expected increased fasting blood sugar but also increased postprandial values. Despite the absence of other risk factors (healthy, BMI 23.9 kg/m², family history bland), we suspected a gestational diabetes component. In addition to nutritional advice, we established a treatment with basal and bolus insulin (NovoRapid and Levemir) in 18th week of pregnancy. Due to fluctuating blood sugar levels, a switch from Levemir to Tresiba was initiated in the 28th week of pregnancy and led to a stabilisation. From week 26, the CGM Dexcom G6 was used for monitoring. With a total daily dose of about 50 units of insulin (50 % bolus, 50% basal), we reached a time in range (3.9 – 8 mmol/l) of 75% with an acceptable number of hypoglycaemias (2 % between 3.0 and 3.9 mmol/l) during the third trimester. In the beginning, the patient did not tolerate well the normal (but for her lower) fasting glucose levels and experienced nausea and headaches, which fortunately improved with time.

Fetal growth was normal, and a healthy baby girl was born at 40 0/7 weeks with a weight of 3.33 kg (P37), a size of 49 cm and APGAR scores of 10/10/10. Postnatal adaptation and alimentation were good, blood glucose was normal. DNA was extracted from cord blood to validate NIPD-M results.

Family 6. Carrier fetus

This was the second pregnancy of a 28-year-old woman diagnosed with MODY at the age of 12 years. Genetic testing showed the heterozygous pathogenic variant c.106C>T, p.Arg36Trp in *GCK*. NIPD-M was performed at 15 weeks pregnancy and showed that the fetus had inherited the familial *GCK* mutation. The patient did not receive insulin treatment but was carefully monitored. Glycemia measurements at wake-up were between 5mmol/l and 6 mmol/l and postprandial glycemia were <8mmol/l. Fetal growth was monitored, and labor was induced at 38 2/7weeks.

The patient delivered a healthy boy, APGAR 10/10/10, pH 7.22 with birth weight 3770g (P75-90), length 51cm (P25-50), HC 36.5cm (P75-90). The boy is now 6 months old and perfectly healthy.

Family 7. Wild-type fetus

The patient was a 27-year-old woman, diagnosed with impaired glucose tolerance at aged 14. There was a family history of diabetes affecting her father, paternal grandmother, and great-grandfather. Her OGTT results (T0=7.6 mmol/l, 2h=11.7 mmol/l) was consistent with MODY-GCK. A molecular test demonstrated that she is heterozygous for *GCK*:c.608T>C, p.Val203Ala mutation. NIPD-M was performed at 16 weeks. Following the results, a treatment with Lantus was initiated (6U, to be increased), and her pregnancy is still ongoing.

**2. DETAILED METHODS**

Panel design

98 SNPs were selected throughout a 0.7 Mbase genomic region surrounding the *GCK* gene, based on a rate of heterozygosity >0.3 in the general population. We selected these SNPs from different sources (gnomAD, 1000-genomes phase 3, and Illumina Omni microarray data) and attempted to cover a large range of heterozygosity rates (0.3 to 0.5), to limit the risk that all SNPs may come from the same individuals, i.e. belong to the same common GCK haplotype.

Additionally, the panel included the *GCK*:p.Gly246Arg mutation, present in family 1.

A QIASeq Targeted DNA custom panel featuring molecular barcodes (Qiagen) was designed to target 50 bp regions encompassing each SNP.

Samples and DNA extraction

Genomic DNA was extracted from venous blood or cord blood using the DNeasy blood and tissue kit and a Qiacube robot (Qiagen). Maternal venous blood was collected into Streck BCT tubes (Streck) between 12 weeks and 30 weeks amenorrhea. When needed, a 500 µl aliquot of blood was withdrawn to extract genomic DNA as above. Plasma was prepared by centrifugation for 10 min at 1'600 x g, 4°C, collected, centrifuged 10 min at 16'000 x g, 4°C and occasionally stored at -80°C. Circulating cell-free DNA was extracted from plasma with the QIAamp MinElute ccfDNA kit (Qiagen) according to manufacturer's instructions, and quantified with a Qubit fluorimeter (Molecular Probes).

Library construction

QIASeq custom targeted libraries were built from 40 ng genomic DNA, using the manufacturer's protocol for genomic DNA and from 7.2 to 50 ng ccfDNA, using the circulating DNA protocol. Libraries were quantified by fluorimetry, and size profiles were verified with a Fragment Analyzer (Agilent).

Sequencing

Samples were pooled and sequenced as 2 x 75 nucleotides with a NextSeq500 sequencer (Illumina). For each sample, 4 to 5 million reads were obtained.

Analysis

Qiagen smCounter2 pipeline (Xu et al. Bioinformatics, 2019) was used to align and filter reads, deduplicate barcodes and build consensus reads using Fgbio subroutines (https://github.com/fulcrumgenomics/fgbio). Allele counts were extracted from the resulting BAM files with bam-readcount (https://github.com/genome/bam-readcount).

The genotypes of the parents and a prior child (if available, otherwise the maternal grandparents) were deduced from allelic counts and parental haplotypes were assembled.

RHDO was performed in Excel, as described by Lo *et al*. (*Sci Translat Med* 2010). Alpha and beta SNPs located upstream and downstream of the mutation were analyzed separately to detect the unlikely occurrence of meiotic recombination within the region of interest. Once this possibility was excluded, a global likelihood ratio was calculated for all alpha SNPs or for all beta SNPs, depending on which yielded the highest statistical significance.

Reducing fetal fraction

A custom filter was introduced in the smCounter2 pipeline to randomly reject a given fraction of molecules. This pipeline begins by gathering reads that share the same barcode and map at the same approximate location (indicating that they originate from the same DNA molecule), then several built-in quality checks can reject a read or a barcode. An extra filter was added at this step, to discard an arbitrarily chosen fraction of barcodes, despite good quality scores.

In script umi_mark.py, two identical lines with the following statement:

handleOneMolecule(alignments, fileout1, fileout2)

were modified as:

if counter%downsampling == 0:

handleOneMolecule(alignments, fileout1, fileout2)

counter += 1

Where *counter* is initialized upon entry and incremented for each new barcode, and *downsampling* was set to 1 to disable filtering or to a larger N value to accept only 1 read out of N.

For data mixing experiments, BAM files from plasma sequencing data and maternal sequencing data, possibly downsampled, were merged in various proportion with samtools merge. The lists of primers detected by the pipeline was merged with a “cat” command and the smCounter2 pipeline was resumed at the primer clip stage.

**3. SUPPLEMENTARY FIGURE LEGENDS**

**Supplementary figure 1. Types of SNPs and their usage**

A and B represent 2 alleles of a given SNP. The bar graphs illustrate allelic frequencies in maternal plasma, with maternal ccfDNA in dark blue and fetal cffDNA in light blue (indistinguishable by sequencing).

Type-1 SNPs: The parents are homozygous for different alleles; the fetus is necessarily heterozygous. Fetal fraction is twice the allelic frequency of the paternal allele in maternal plasma.

Type-2 SNPs: Both parents are homozygous for the same allele. These SNPs can be used to appraise sequencing noise, although this can also be achieved with any invariant position in the vicinity of other SNPs.

Type-3 SNPs: The father is heterozygous and the mother homozygous. This type is divided into alpha SNPs, when the mother is homozygous for the allele corresponding to paternal haplotype 1, and beta SNPs when it’s paternal haplotype 2. These SNPs are only needed in case of a biparental mutation, to detect which paternal haplotype was transmitted to the fetus, or when there isn’t enough type-1 SNPs to determine FF. The presence of a paternal-specific allele in maternal plasma indicates that the corresponding paternal haplotype was transmitted to the fetus.

Type-4 SNPs: The mother is heterozygous and the father homozygous. This type is divided into alpha SNPs, when the father is homozygous for the allele corresponding to maternal haplotype 1, and beta SNPs when it’s maternal haplotype 2. These SNPs are used to appraise the balance of maternal haplotypes, which is biased by the presence of fetal DNA and forms the basis of RHDO analysis (see supplementary figure 2 for details).

Type-5 SNPs: Both parents are heterozygous, the fetus could be heterozygous or homozygous for either allele. These SNPs can be used in consanguineous couples, when RHDO is impossible due to lack of type-4 SNPs (see supplementary figure 2).

**Supplementary figure 2. Principles of RHDO and RGDO**

Upper panel: RHDO uses type-4 SNPs, which are heterozygous in the mother and homozygous in the father, to determine which maternal haplotype is overrepresented in maternal plasma. For alpha SNPs, the paternal allele corresponds to maternal haplotype 1, which is overrepresented if the fetus inherited maternal haplotype 1. Conversely, haplotype 2 is overrepresented for beta SNPs if the fetus received maternal haplotype 2.

Lower panel: RGDO uses type-5 SNPs which are heterozygous in both parents, thus the individual contribution of each parent cannot be ascertained. However, if there are only 2 haplotypes in the couple, which can happen when there are several loops of consanguinity in the family, fetal genotype can be directly determined from the balance of haplotypes in maternal plasma (see Fokstuen et al*. Clin. Genet*. 2023 for details).

**Supplementary figure 3. Analytical workflow**

**SETUP**: Parents are genotyped for all SNPs in the panel and haplotypes (colored bars) are reconstructed using a first-degree relative, preferably a prior conceptus of the couple. If none is available, phasing can be achieved via long-read sequencing for the parent carrying the mutation.

**NIPD-M**: ccfDNA is extracted from maternal plasma. Depending on the presence of a stretch of biallelic identity-by-descent (or identity-by-state), different SNP types are available. Type-1 SNPs (parents homozygous for different alleles) are used to determine FF, type-3 SNPs (father heterozygous, mother homozygous) to determine the paternal haplotype in case of a biparental mutation, and type-4 SNPs (mother heterozygous, father homozygous) to determine the maternal haplotype via RHDO analysis. In a stretch of identity, only type-5 SNPs (both parents heterozygous) exist. These can be leveraged for RGDO analysis, whereas FF can be determined with type-1 SNPs located outside the stretch of identity (e.g. using a pangenomic SNP panel).

**Supplementary figure 4. Crossing-over detection**

Heterozygous SNPs (diamonds) are tallied by order of appearance while moving away from the mutation in either direction. For each SNP, molecular counts from this and all preceding SNPs are cumulated in the calculation of a haplotype ratio: H1 / (H1+H2).

These SNPs are plotted in a bidirectional graph, with the number of molecules tallied in the divergent X-axes and the haplotype ratio in the common Y-axis. Expected ratios differ for alpha and beta SNPs, which must be plotted separately.

Colored curves represent the diagnostic threshold (by convention 1200:1) and depend only on the fetal fraction (FF) and the number of molecules tallied (N, x-axis).

The haplotype inherited by the fetus is ascertained once cumulated counts pass one of the threshold curves (thick arrows). To exclude a crossing-over, the same haplotype must be found on both sides of the mutation (H1 in this example). Although the theoretical probability of a double crossing over is not null, crossover interference effectively prevents two recombination events from occurring in such close vicinity.

**Supplementary figure 5. Calculations when crossing-over is not excluded**

When the maternal haplotype transmitted to the fetus can only be ascertained on one side of the mutation, a crossing-over cannot be excluded. The probability of crossing-over, P_CO,_ is the product of the distance between the mutation and the first informative SNP on the conclusive side (159 kb in this example), multiplied by the recombination rate at the *GCK* locus (1.1 cM/Mb). This probability is combined with the likelihood for the opposite haplotype on the inconclusive side of the mutation, P_H2_ (1/41 here), into the joint probability that haplotype 2 is present at the mutation site in the fetus, P_CO+H2_. This example is from family 4.
