# Supplementary material for: Enhancing fetal outcomes in GCK-MODY pregnancies: a precision medicine approach via non-invasive prenatal GCK mutation detection
Source: Front Med (Lausanne). 2024 Apr 30;11:1347290. doi: 10.3389/fmed.2024.1347290 (PMC11091329; doi:10.3389/fmed.2024.1347290)
Supplement: Supplementary Figure 1 — Type of SNPs and their usage. [file Image_1.pdf]

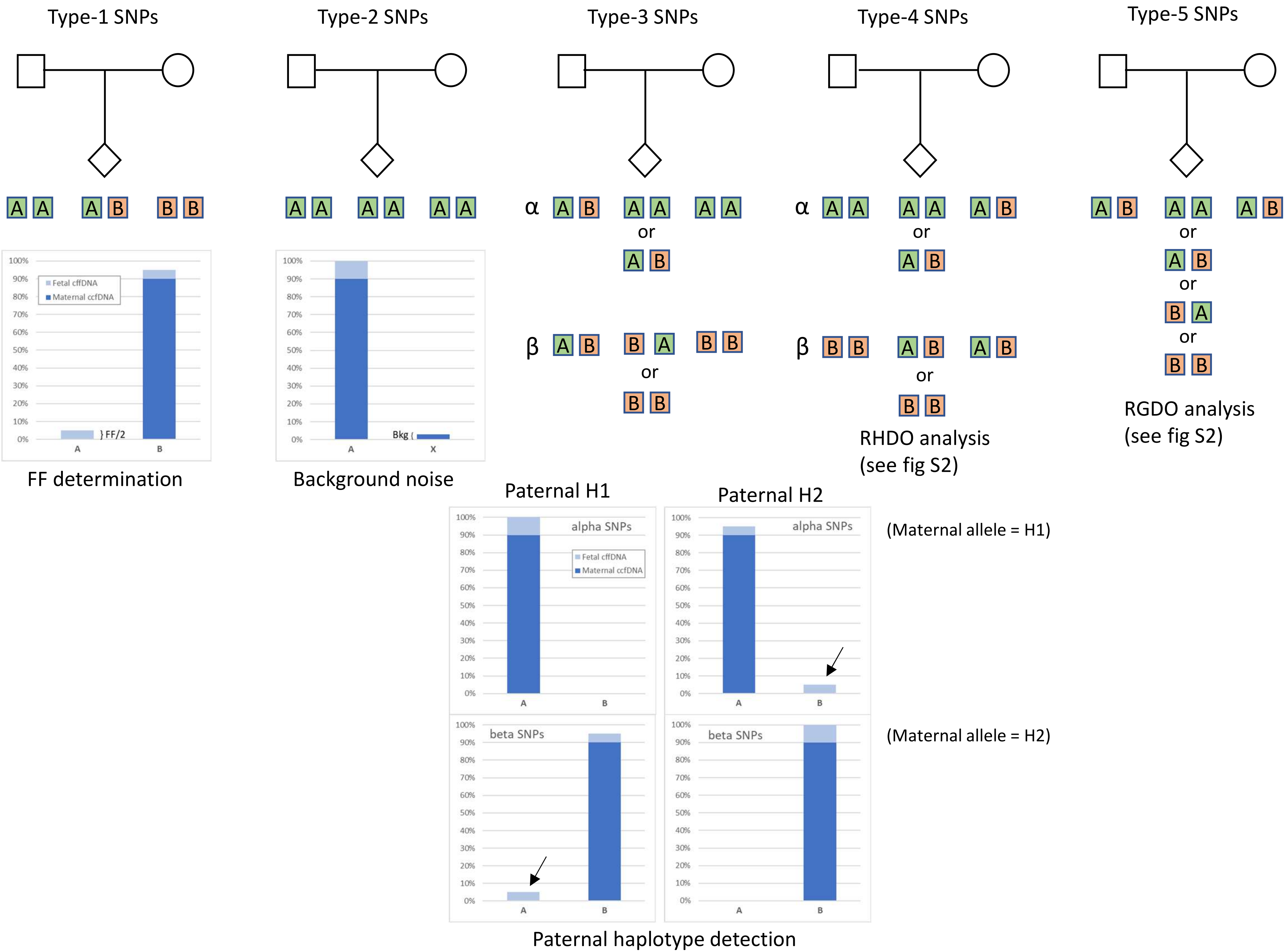

Type-1 SNPs
Type-2 SNPs
Type-3 SNPs
Type-4 SNPs
Type-5 SNPs
A A
A B
B B
A A
A A
A A
α
A B
A A
or
A B
A A
β
A B
B A
or
B B
B B
α
A A
A A
or
A B
A B
β
B B
A B
or
B B
A B
A B
A A
or
A B
or
B A
or
B B
A B
Fetal cffDNA
Maternal ccfDNA
} FF/2
A
B
FF determination
Bkg {
A
X
Background noise
RHDO analysis
(see fig S2)
RGDO analysis
(see fig S2)
Paternal H1
Paternal H2
alpha SNPs
beta SNPs
(Maternal allele = H1)
(Maternal allele = H2)
Paternal haplotype detection
