# Supplementary material for: Enhancing fetal outcomes in GCK-MODY pregnancies: a precision medicine approach via non-invasive prenatal GCK mutation detection
Source: Front Med (Lausanne). 2024 Apr 30;11:1347290. doi: 10.3389/fmed.2024.1347290 (PMC11091329; doi:10.3389/fmed.2024.1347290)
Supplement: Supplementary Figure 2 — Principles of RHDO and RGDO. [file Image_2.pdf]

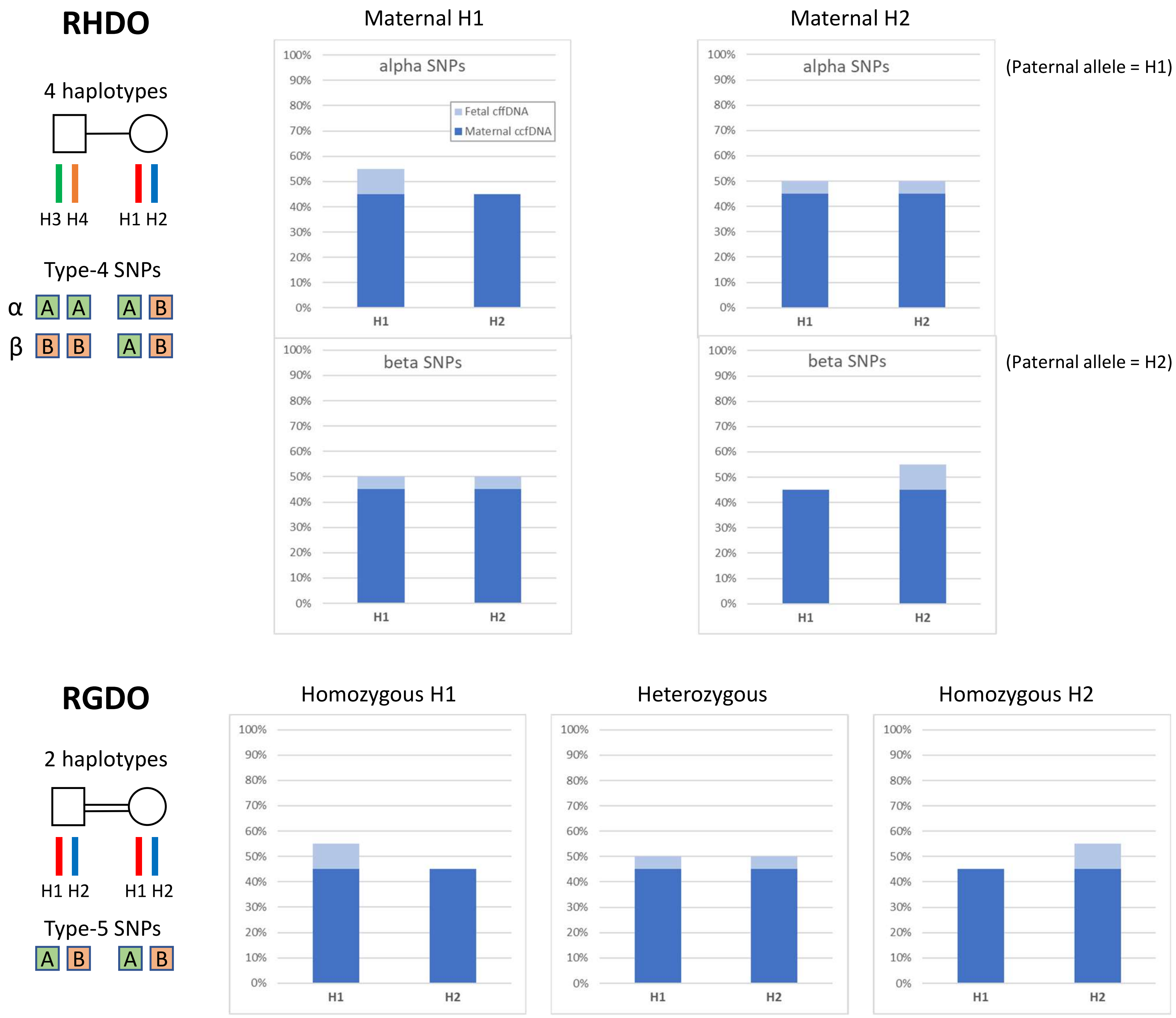
RHDO
4 haplotypes
H3 H4
H1 H2
Type-4 SNPs
α A A A B
β B B A B
Maternal H1
Maternal H2
alpha SNPs
beta SNPs
Fetal cffDNA
Maternal ccfDNA
(Paternal allele = H1)
(Paternal allele = H2)
RGDO
2 haplotypes
H1 H2
H1 H2
Type-5 SNPs
A B A B
Homozygous H1
Heterozygous
Homozygous H2
0%
10%
20%
30%
40%
50%
60%
70%
80%
90%
100%
H1
H2
