# Supplementary figures and images for: Enhancing fetal outcomes in GCK-MODY pregnancies: a precision medicine approach via non-invasive prenatal GCK mutation detection
Source: Front Med (Lausanne). 2024 Apr 30;11:1347290. doi: 10.3389/fmed.2024.1347290 (PMC11091329; doi:10.3389/fmed.2024.1347290)

## SETUP

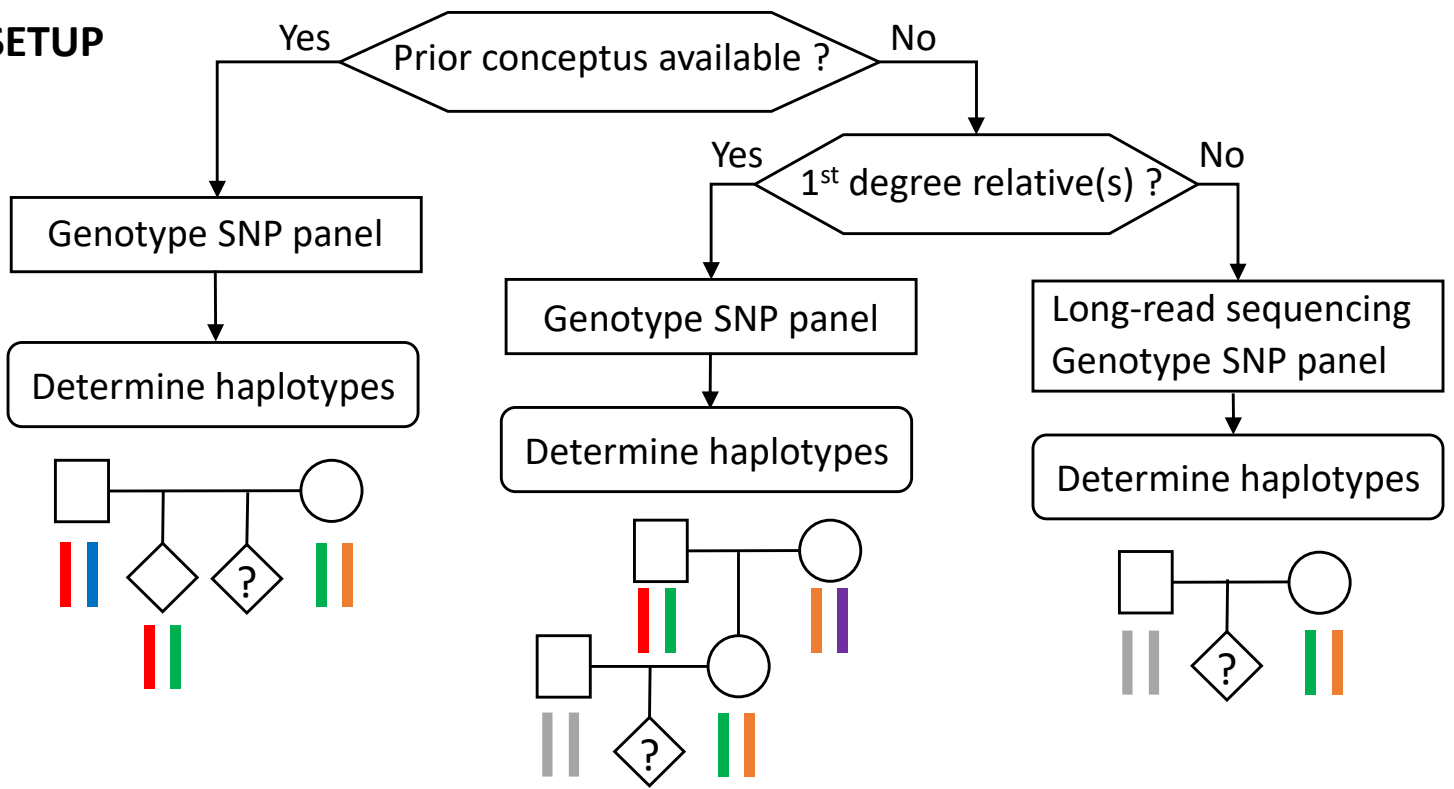

## NIPD-M

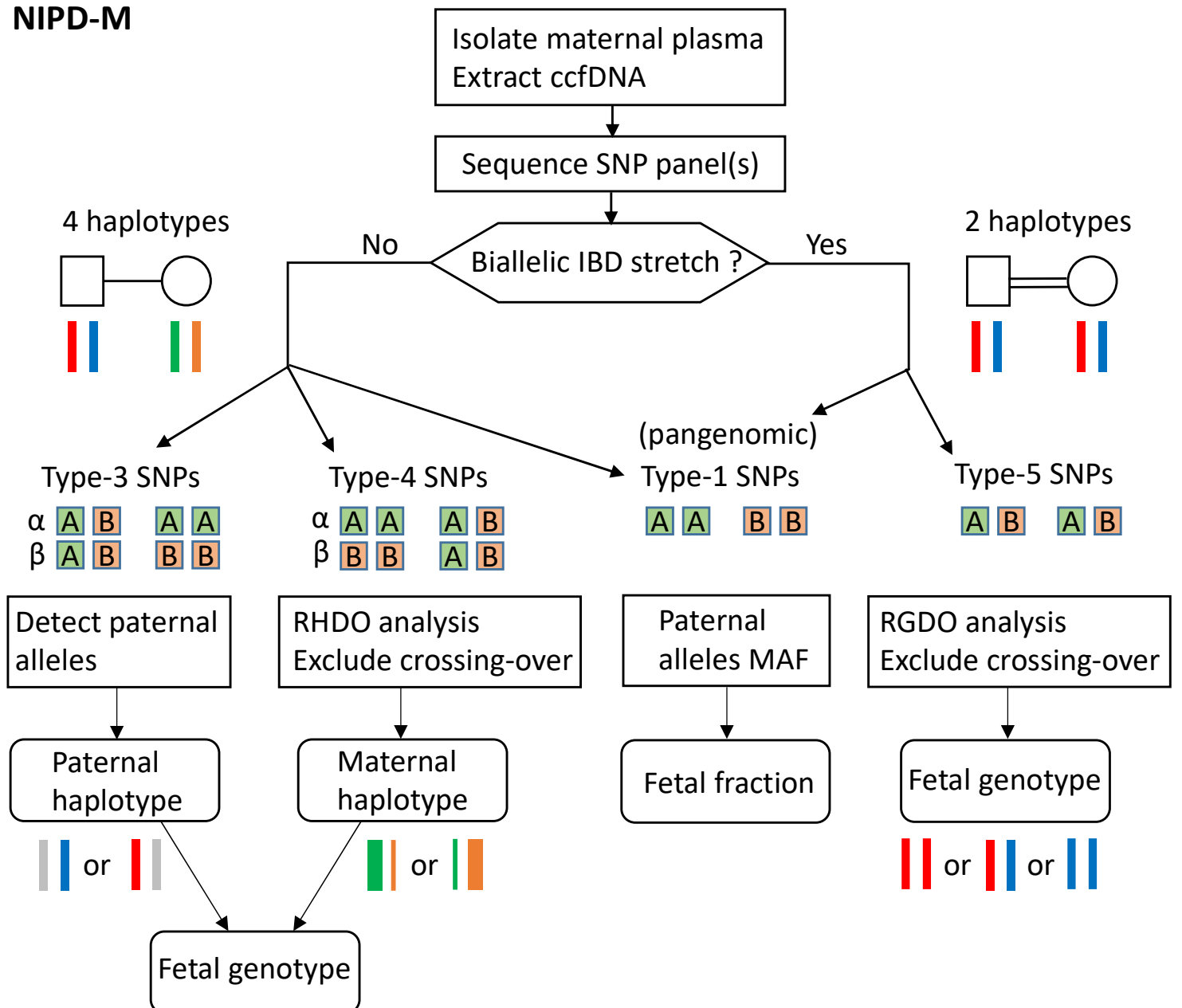

Supplement: Supplementary Figure 3 — Analytical workflow. [file Image_3.pdf]

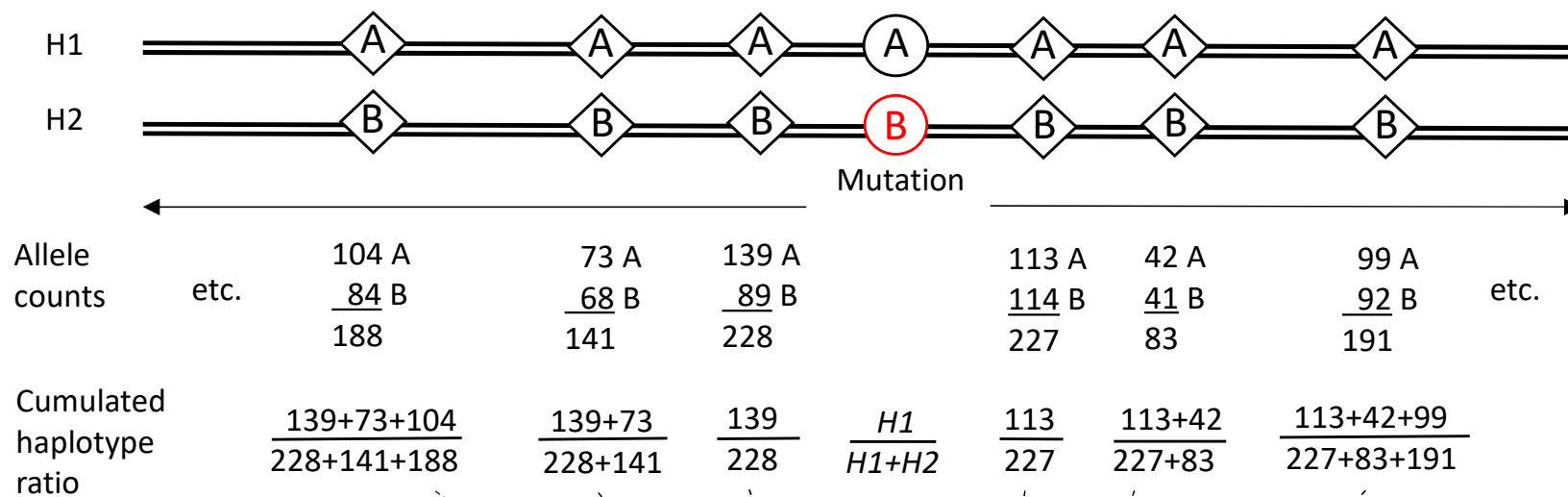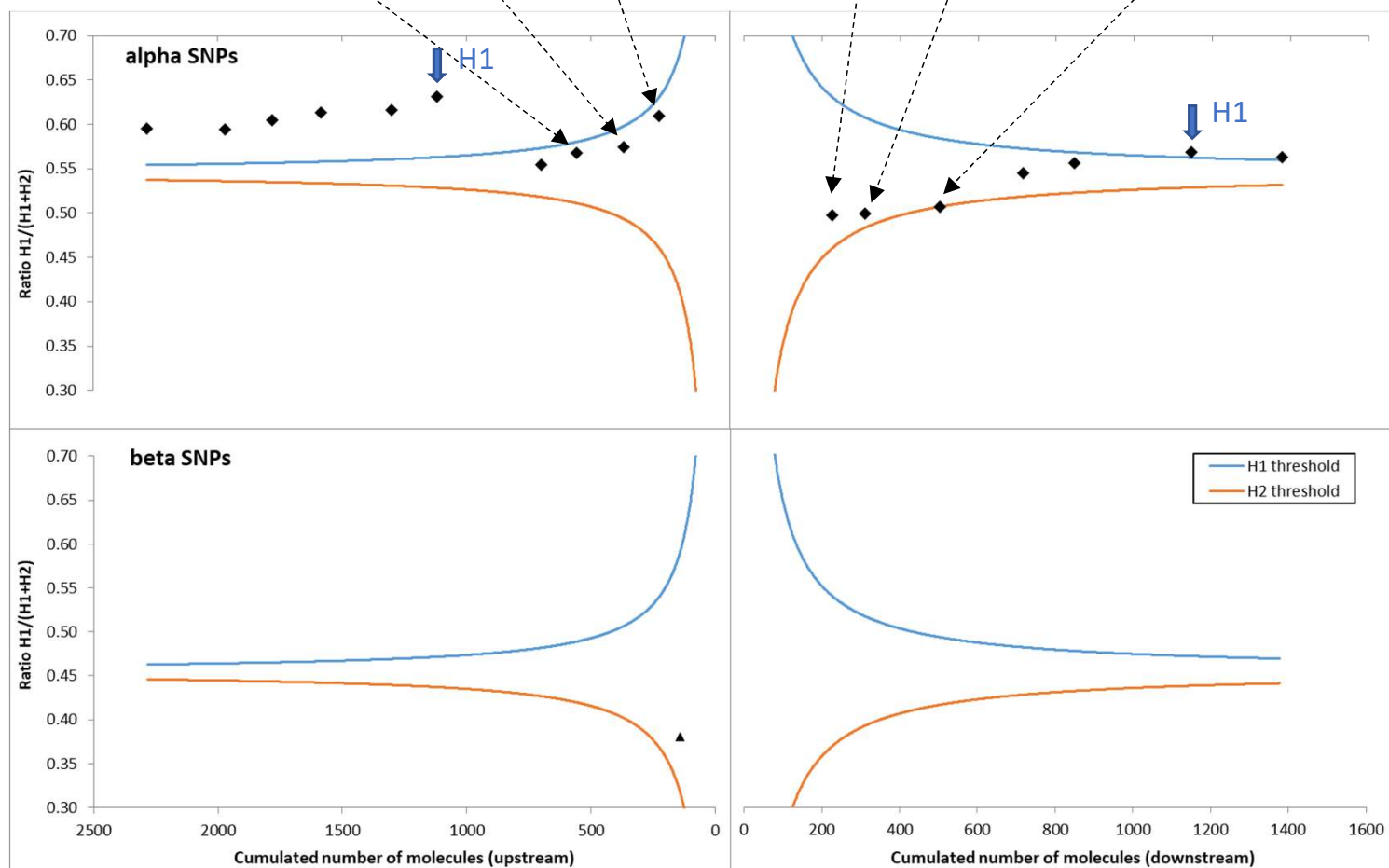

$$\frac{\ln(1200)/N - \ln(1-FF)}{\ln(1+FF) - \ln(1-FF)}$$

$$\frac{-\ln(1200)/N - \ln(1-FF)}{\ln(1+FF) - \ln(1-FF)}$$

$$\frac{\ln(1200)/N + \ln(1+FF)}{\ln(1+FF) - \ln(1-FF)}$$

$$\frac{-\ln(1200)/N + \ln(1+FF)}{\ln(1+FF) - \ln(1-FF)}$$

Supplement: Supplementary Figure 4 — Crossing-over detection. [file Image_4.pdf]

Recombination rate = 1.1 cM/Mb

$$159 \text{ kb} \rightarrow P_{CO} = 0.159 * 1.1\% = 0.175\%$$

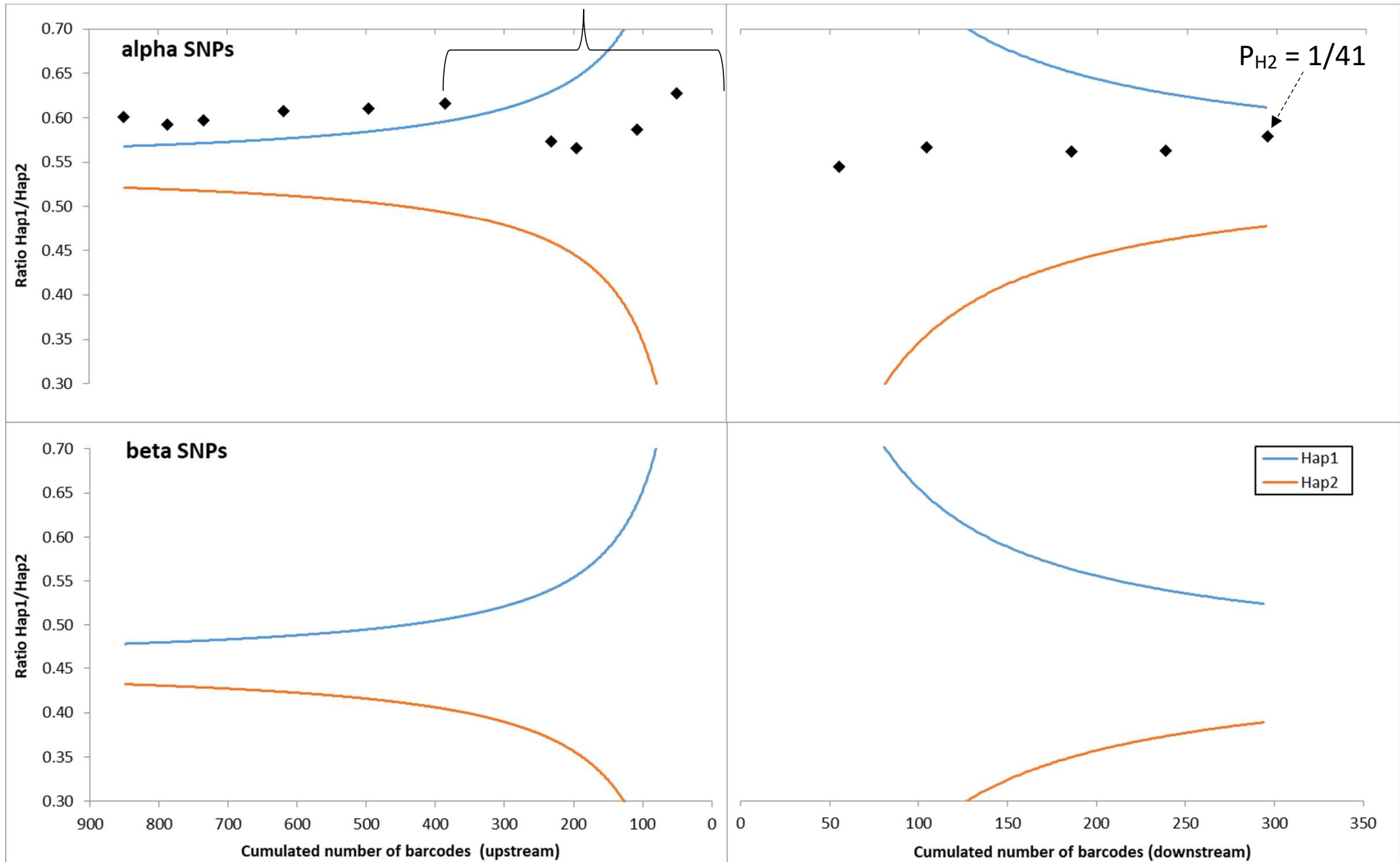

$$\text{Joint probability: } P_{CO+H2} = 0.175\% * 1/41 = 1/23442$$

Supplement: Supplementary Figure 5 — Calculations when crossing-over is not excluded. [file Image_5.pdf]
